# Lack of Phenotypical and Morphological Evidences of Endothelial to Hematopoietic Transition in the Murine Embryonic Head during Hematopoietic Stem Cell Emergence

**DOI:** 10.1371/journal.pone.0156427

**Published:** 2016-05-26

**Authors:** Kazuhide Iizuka, Tomomasa Yokomizo, Naoki Watanabe, Yosuke Tanaka, Motomi Osato, Tomoiku Takaku, Norio Komatsu

**Affiliations:** 1 Department of Hematology, Juntendo University School of Medicine, Tokyo, Japan; 2 International Research Center for Medical Sciences, Kumamoto University, Kumamoto, Japan; 3 Laboratory of Stem Cell Biology, Center for Developmental Biology, RIKEN Kobe, Kobe, Japan; 4 Cancer Science Institute of Singapore, National University of Singapore, Singapore, Singapore; 5 Department of Paediatrics, National University of Singapore, Singapore, Singapore; Johns Hopkins School of Medicine, UNITED STATES

## Abstract

During mouse ontogeny, hematopoietic cells arise from specialized endothelial cells, i.e., the hemogenic endothelium, and form clusters in the lumen of arterial vessels. Hemogenic endothelial cells have been observed in several embryonic tissues, such as the dorsal aorta, the placenta and the yolk sac. Recent work suggests that the mouse embryonic head also produces hematopoietic stem cells (HSCs)/progenitors. However, a histological basis for HSC generation in the head has not yet been determined because the hematopoietic clusters and hemogenic endothelium in the head region have not been well characterized. In this study, we used whole-mount immunostaining and 3D confocal reconstruction techniques to analyze both c-Kit^+^ hematopoietic clusters and Runx1^+^ hemogenic endothelium in the whole-head vasculature. The number of c-Kit^+^ hematopoietic cells was 20-fold less in the head arteries than in the dorsal aorta. In addition, apparent nascent hematopoietic cells, which are characterized by a “budding” structure and a Runx1^+^ hemogenic endothelium, were not observed in the head. These results suggest that head HSCs may not be or are rarely generated from the endothelium in the same manner as aortic HSCs.

## Introduction

Hematopoietic stem cells (HSCs) and progenitors arise from several anatomically distinct regions during development [1–3]. Many studies have described the importance of the aorta-gonad-mesonephros (AGM) region and have revealed that clusters of hematopoietic cells are observed in the lumen of the dorsal aorta at the time of HSC generation [4–8]. Because cells within hematopoietic clusters express cell-surface markers, such as c-Kit and CD31, these cells can be isolated using a fluorochrome-conjugated antibody and flow cytometry. The cluster-enriched population exclusively retains the long-term repopulation ability in lethally irradiated adult mice, suggesting that HSCs form within these hematopoietic clusters [9]. These cluster cells are generated by the transdifferentiation of hemogenic endothelial cells, a process known as endothelial-hematopoietic transition (EHT) [10–13].

The hemogenic potential of endothelial cells has been documented in several embryonic tissues, such as the AGM, the yolk sac, the placenta and the endocardium [13–17]. Endothelial cells isolated from these tissues give rise to definitive types of hematopoietic cells *in vitro*. The transcription factor Runx1, which is expressed by both hematopoietic cluster cells and endothelial cells[18, 19], has been shown to be required in hemogenic endothelial cells during mid-gestation for the generation of definitive HSCs/progenitors and for the formation of hematopoietic clusters [18, 20, 21].

Recent work has suggested that the mouse embryonic head also produces HSCs/progenitors [22]. The long-term repopulation capacity has been detected in the head at embryonic day (E) 10.5, which is when HSCs first appear in the AGM region. In addition, a lineage-tracing experiment using SP-A Cre transgenic mice, which specifically express Cre recombinase in the embryonic head endothelium, demonstrated that SP-A^+^ cell-derived progenies physiologically contribute to the adult HSC population. However, because the head has not been recognized as a hemogenic site, few histological studies have examined hematopoietic cluster distribution, and a comprehensive picture of this process is lacking [22–24].

We previously developed a whole-mount immunostaining technique to visualize hematopoietic clusters within blood vessels, and we reported the cartography of c-Kit^+^ hematopoietic clusters in the dorsal aorta, vitelline artery, and umbilical artery [9]. Using a similar methodology in this study, we extensively examined whole-mount embryonic head tissues to determine the localization of hematopoietic clusters. Unexpectedly, we did not find visual evidence of EHT structures in the head. Moreover, Runx1-GFP, a marker of hemogenic endothelium, is not expressed in the head vasculature. These results suggest that embryonic head HSCs may not be or are rarely generated from the hemogenic endothelium in the same manner as aortic HSCs.

## Materials and Methods

### Mice and embryos

*Runx1-GFP* (*eR1-GFP*) transgenic mice and *Runx1-EnSA-MerCreMer* (*Runx1-SACre*) mice were described previously [25, 26]. Embryos were generated via the timed mating of C57BL/6 males and females, *eR1-GFP* transgenic males and C57BL/6 females, and *Runx1*^*SACre/+*^ males and *ROSA26*^*YFP/YFP*^ females. The embryos were staged according to the embryonic day, somite pairs (sp) and Thelier criteria (http://genex.hgu.mrc.ac.uk/intro.html). The hydroxytamoxifen (4-OHT) injection protocol was described previously [27]. All animal procedures were approved by the Ethics Committees on Animal Experimentation, Juntendo University (Approval numbers: 240175, 250153, 260144).

### Embryo dissection and whole-mount immunostaining

The caudal half was prepared as described previously [28]. The head was incised at the median line to prepare two sagittal blocks before immunostaining. Whole-mount immunostaining was performed as described previously [28]. Primary antibodies to c-Kit (2B8, BD Biosciences or polyclonal goat IgG, R&D), CD45 (30-F11, BD Biosciences), and biotinylated anti-CD31 (MEC 13.3, BD Biosciences) were used in this study. The secondary antibodies (or streptavidin conjugates) used in this study were goat anti-rat IgG-Alexa647 (Invitrogen), Cy3-streptavidin (Jackson ImmunoResearch), Alexa488-streptavidin (Invitrogen), goat anti-rat IgG-Alexa555 (Invitrogen) and donkey anti-goat IgG-Dylight649 (Jackson ImmunoResearch). GFP and YFP were detected with rabbit anti-GFP antibodies (MBL), followed by goat anti-rabbit IgG-Alexa647 (Invitrogen).

### Confocal microscopy and image analysis

The immunostained embryos were mounted in a 1:2 mix of benzyl alcohol and benzyl benzoate (BABB) to increase the transparency of the tissues; they were then analyzed with a confocal microscope (Zeiss LSM 510 Meta, Zeiss LSM 700, EC Plan-Neofluar 10x/NA 0.3, Plan-Neofluar 20x/NA 0.5). Tile scanning was performed using a motor-driven x-y scanning stage and the MultiTime Macro function in the LSM510 software or Zeiss Zen. Three-dimensional reconstructions were generated from *z*-stacks with the LSM Image Browser (Zeiss) or Imaris software (Bitplane). Arteries and veins were identified by tracing major vessels, such as the internal carotid artery and the anterior cardinal vein. Small vessels less than 12 μm diameter were defined as capillaries [29].

### Cell preparation and flow cytometry

Single-cell suspensions were prepared by treating tissues with collagenase [0.125% in phosphate-buffered saline (PBS)/10% fetal calf serum (FCS)/1% penicillin/streptomycin] for 1 hour at 37°C. The cells were stained with the following: APC-anti-c-Kit (2B8, BD Biosciences), PE-anti-CD31 (MEC13.3, BD Biosciences), PerCP-Cy5.5-anti-SSEA1 (MC-480, eBioscience), APC-Cy7-anti-CD45 (30-F11, BD Biosciences), or Alexa488-NGF receptor (Advanced Targeting Systems). The cells were analyzed using FACS Fortessa (BD Biosciences).

## Results

### Whole-head scanning of c-Kit^+^ cells in the mouse embryo

**Fig 1:**
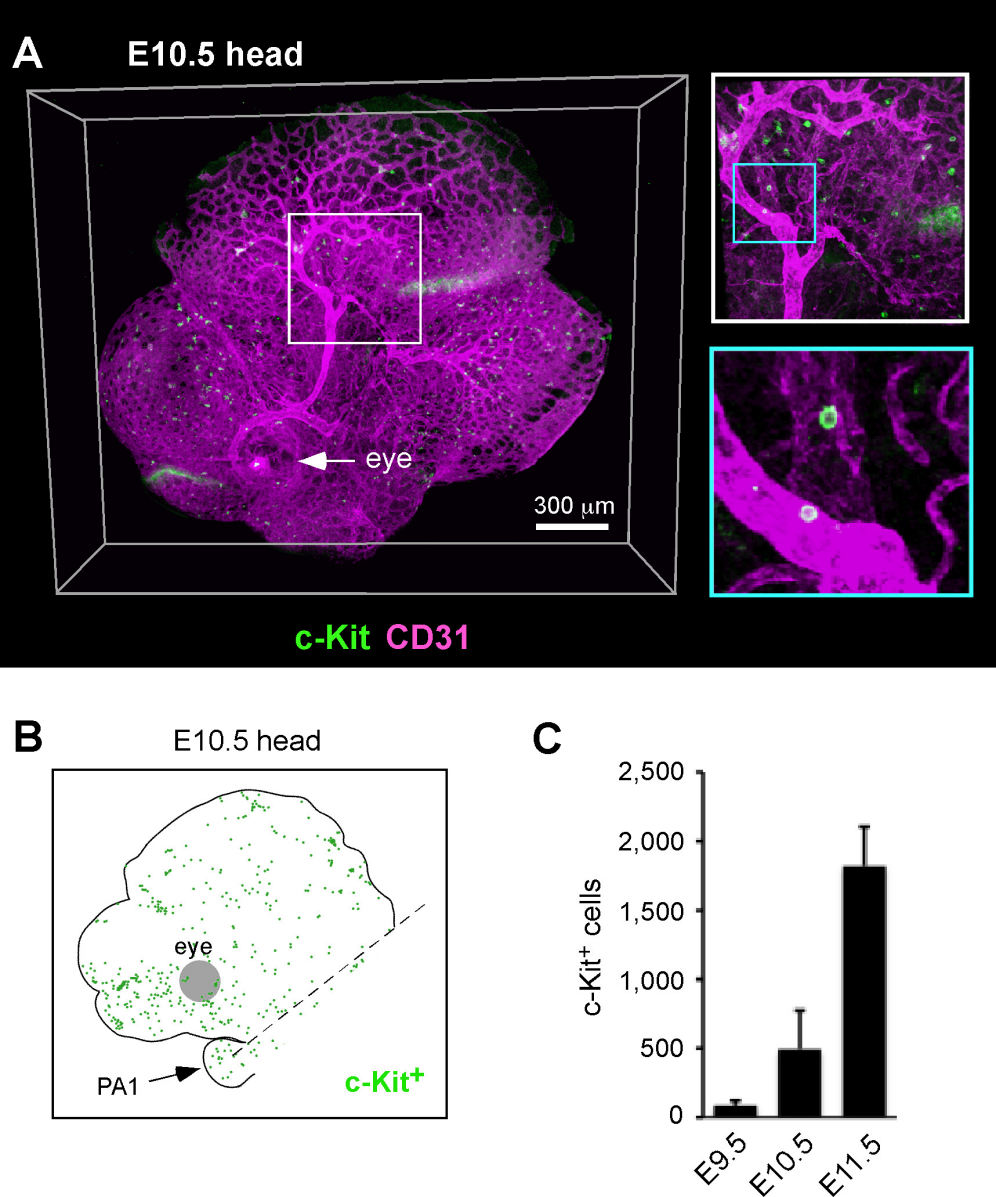
Three-dimensional analysis of c-Kit^+^ cells in the embryonic head. (A) 3D confocal image of c-Kit (green) and CD31 (magenta) expression in the mouse head region at E10.5 (35 sp). The whole-head image was acquired using tile scanning (20 tiles). (B) Cartographic distribution of c-Kit^+^ cells in the head. The c-Kit^+^ cells observed in the left half of the head are plotted. The head region for counting c-Kit^+^ cells is indicated by the broken line (above the first pharyngeal arch). PA1: first pharyngeal arch. (C) The number of c-Kit^+^ cells in the head vasculature at different times of development. E9.5 (n = 2, 25 and 26 sp), E10.5 (n = 4, 35 and 36 sp) and E11.5 (n = 4, 45–47 sp) were analyzed.

To examine the precise localization and quantity of hematopoietic clusters in the mouse embryonic head, we used whole-mount immunostaining and three-dimensional (3D) confocal microscopic analysis [9]. The whole-mounts were stained with anti-c-Kit and anti-CD31 antibodies to visualize the hematopoietic clusters and endothelial cells, respectively. Because the long-term repopulating ability was detected starting at E10.5 (36 sp) [22], we focused on approximately the E10 stage. Our initial experiment showed that an overnight incubation allowed the antibody to sufficiently diffuse to an adequate depth in the E10.5 whole-mount embryos (S1 Fig). To ensure the complete penetration of antibodies, the head was incised at the median line to prepare two sagittal blocks before immunostaining. The stained and cleared heads were scanned using a 20x objective. Over 5,000 images of E10.5 embryos acquired from 20 hours of tile-scanning were reconstructed and stitched to generate a comprehensive 3D picture of the immunostained head (Fig 1A). This method allowed us to detect all of the c-Kit^+^ cells in the head region at single-cell resolution (Fig 1A).

In addition to being observed inside blood vessels, many c-Kit^+^ cells were observed in the mesenchyme (Fig 1A). These cells were negative for hematopoietic (CD45) and endothelial (CD31) markers and positive for the p75 neutrophin receptor (S2 Fig), suggesting that they are neural crest-derived cells [30]. Therefore, mesenchymal c-Kit^+^ cells were excluded from our analysis. The c-Kit^+^ cells were scattered over the entire head vasculature (Fig 1B). The number of c-Kit^+^ cells increased between E9.5 and E11.5 (Fig 1C).

### A small number of c-Kit^+^ cells are localized in head arteries

**Fig 2:**
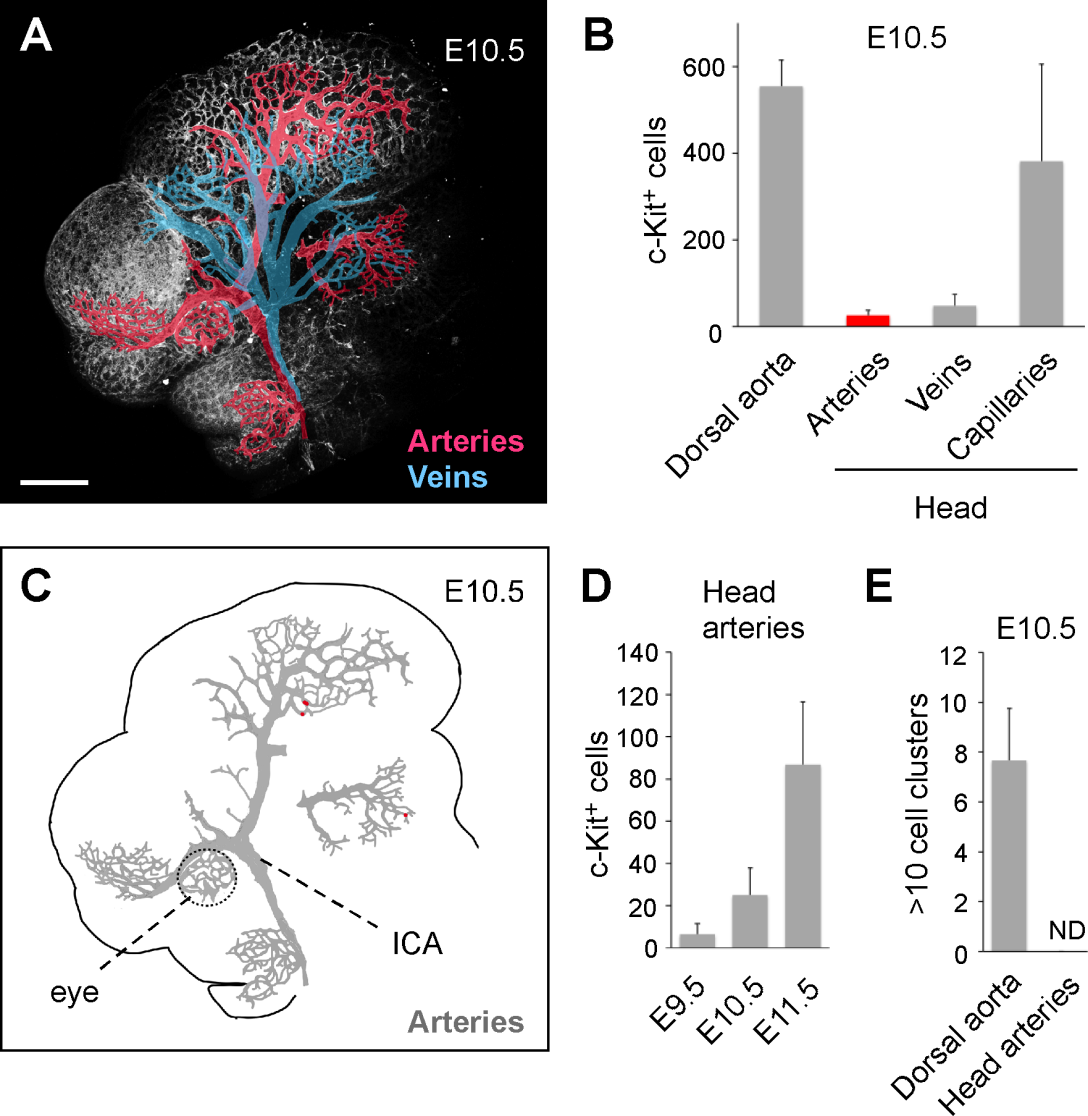
Spatio-temporal quantitation of c-Kit^+^ cells in the head arteries. (A) Illustrative example showing arteries (red) and veins (blue) in the E10.5 (35 sp) head. Scale bar: 300 μm. (B) Number of c-Kit^+^ cells in the dorsal aorta (n = 3) and the head vasculature (n = 4) at E10.5 (35–37 sp). (C) Cartographic distribution of c-Kit^+^ cells in the head arteries. The c-Kit^+^ cells observed in the left half of the head are plotted (red circles). The grey region represents arteries. See S3 Fig for the complete mapping of the c-Kit^+^ cells in the head vasculature. ICA: internal carotid artery. (D) Number of c-Kit^+^ cells in the head arteries at different times of development. E9.5 (n = 2, 25 and 26 sp), E10.5 (n = 4, 35 and 36 sp) and E11.5 (n = 4, 45–47 sp) were analyzed. (E) The number of hematopoietic clusters with more than 10 cells at E10.5 (35–37 sp).

The c-Kit^+^ cells that were observed within the head vasculature could be a mixture of *de novo* generated cells from the head endothelium and circulating cells that originated from other organs. Because hematopoietic clusters have only been observed in arteries and because EHT is closely associated with arterial characteristics [4, 9–12, 17, 31, 32], the c-Kit^+^ cells in the veins and capillaries were likely circulating cells that encountered the vessel walls by chance. To clarify the anatomical locations of c-Kit^+^ cells, we categorized the head blood vessels into three groups: arteries, veins and capillaries (Fig 2A and 2B, S3 Fig). The locations of the c-Kit^+^ cells were then mapped to these groups (S4 Fig). One representative map of c-Kit^+^ cells in E10.5 head arteries is shown in Fig 2C. At E10.5, 555 ± 59 c-Kit^+^ cells were attached to the wall of the dorsal aorta whereas 22-fold fewer cells (25 ± 12 cells) were observed in the head arteries (Fig 2B and 2D). The number of large clusters (clusters containing more than 10 cells) also differed significantly between the dorsal aorta and the head arteries (S5 Fig). At E10.5, large clusters were absent in the head arteries (Fig 2E). Moreover, in contrast to the dorsal aorta [9], a c-Kit^+^ cell-rich area was not found in the head vasculature (S4 Fig).

### Budding structure is not observed at the head arteries

**Fig 3:**
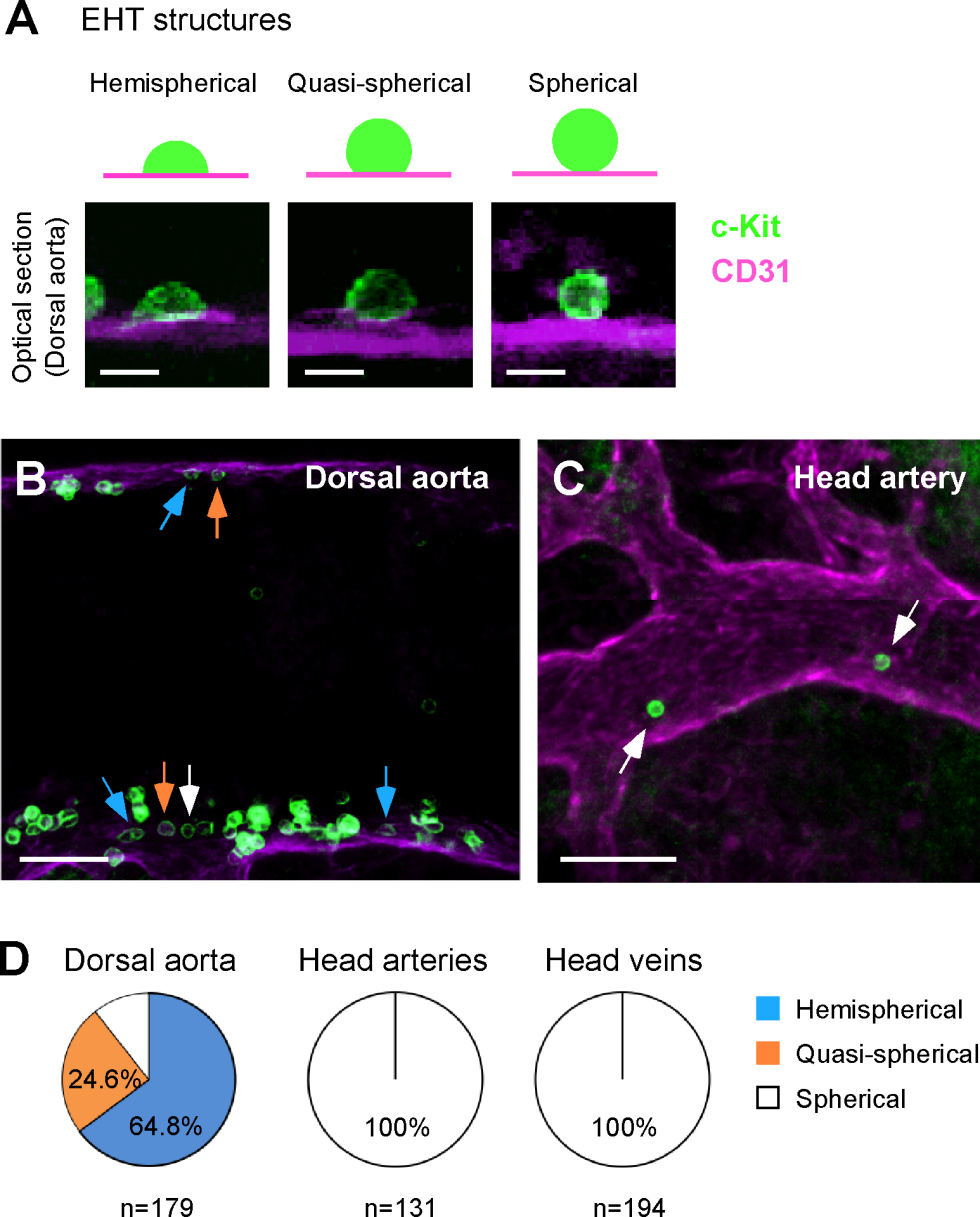
Morphological analysis of c-Kit^+^ cells in the head vasculature. (A) Examples of spherical, quasi-spherical and hemispherical cells localized adjacent to the endothelial layer. (B) c-Kit^+^ cells in the E10.5 (36 sp) dorsal aorta. Blue, orange and white arrows indicate hemispherical, quasi-spherical and spherical cells, respectively. (C) c-Kit^+^ cells in the E10.5 (36 sp) head artery. (D) Frequency of spherical, quasi-spherical and hemispherical c-Kit^+^ cells in the dorsal aorta (n = 179), head arteries (n = 131) and head veins (n = 194). Seven embryos (E10.5, 35–37 sp) were analyzed. Scale bars: 10 μm in A; 50 μm in B and C.

Although a small number of c-Kit^+^ cells were detected in the head arteries, the origin of these cells, e.g., EHT from the head endothelium or migration from other organs, remains unclear. During EHT, the flat hemogenic endothelium undergoes significant morphological changes to generate spherical hematopoietic cells [10–12]. Therefore, if c-Kit^+^ hematopoietic cells are generated *in situ* via EHT, hemispherical or quasi-spherical c-Kit^+^ cells should be transiently observed (Fig 3A, top). Unlike the conventional tissue sectioning method, our whole-mount transparency method almost completely preserves the 3D cellular morphology, which allows us to search for transient structural EHT states in the immunostained embryos (Fig 3A). At the wall of the E10.5 dorsal aorta, which is known to be an active site for EHT, 88% of the c-Kit^+^ cells were hemispherical or quasi-spherical (Fig 3B and 3D). In contrast, only spherical c-Kit^+^ cells were observed in the head arteries and veins (Fig 3C and 3D). Thus, we failed to detect structures indicative of EHT in the head arteries.

### Absence of Runx1-GFP^+^ hemogenic endothelium in the head vasculature

**Fig 4:**
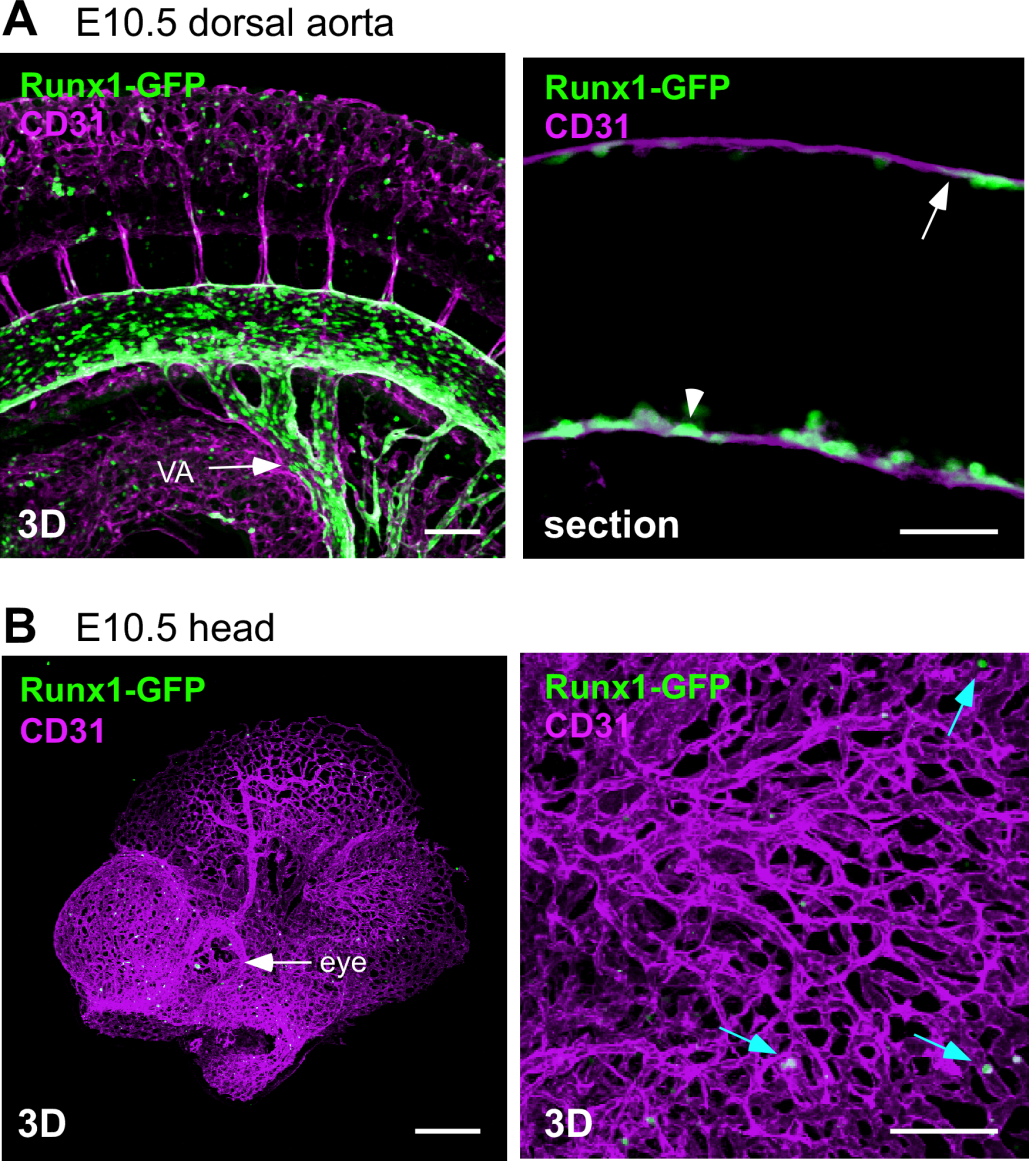
Absence of Runx1-GFP^+^ endothelial cells in the head vasculature. Whole-mount immunostaining of the E10.5 (35 sp) *Runx1-GFP* transgenic mice for GFP (green) and CD31 (magenta) expression. (A) Dorsal aorta. Note that Runx1-GFP is expressed in the flat endothelial cells (arrow) and the hemispherical cells (arrowhead). VA: vitelline artery. Scale bars: 100 μm in the left panel; 50 μm in the right panel. (B) Head. Runx1-GFP was detected in the round cells (blue arrows), but not in the endothelial cells. Scale bars: 300 μm in the left panel; 100 μm in the right panel.

Next, we examined the head vasculature for the presence of hemogenic endothelium. Thus, we utilized *Runx1-GFP* transgenic reporter mice. GFP is expressed in these mice under the control of the *Runx1* +24 mouse conserved noncoding element (eR1), which allows the hemogenic endothelium to be visualized by GFP [25, 33]. As previously reported, Runx1 expression was detected in the middle segment of the dorsal aorta at E10.5 (Fig 4A) [25]. In this region, many flat endothelial cells and hemispherical cells were positive for Runx1-GFP (Fig 4A). In contrast, the head endothelium was negative for Runx1-GFP (Fig 4B). Consistent with the cellular morphology analysis shown in Fig 3, we failed to detect any evidence of EHT in the head.

### Runx1-GFP is downregulated in head c-Kit^+^ cells

**Fig 5:**
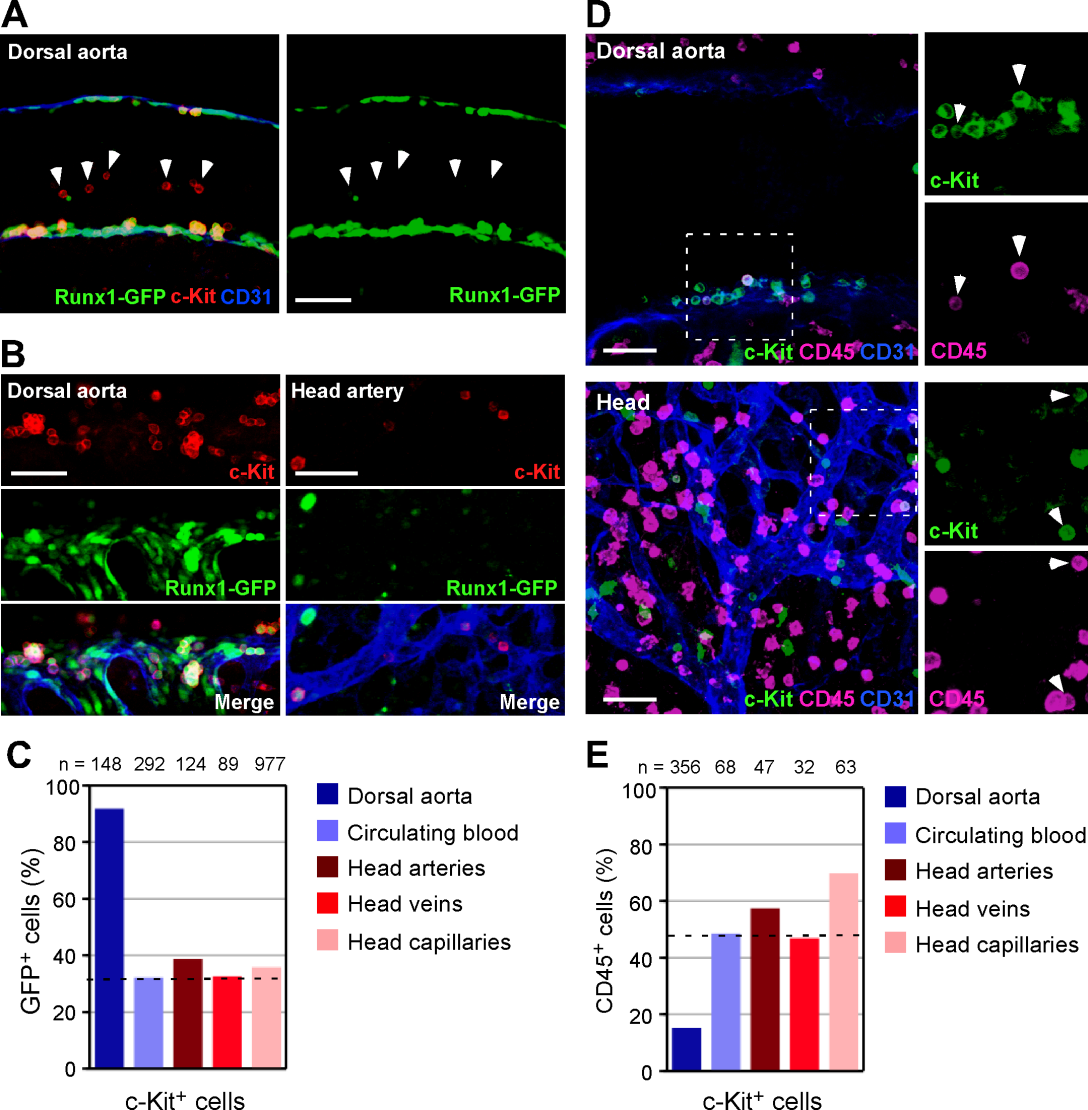
Runx1-GFP and CD45 expression in head c-Kit^+^ cells. (A-C) Whole-mount immunostaining of E10.5 *Runx1-GFP* transgenic mice for GFP (green), c-Kit (red) and CD31 (blue) expression. (A) Sagittal image of the dorsal aorta (35 sp). The c-Kit^+^ circulating cells (arrowheads) were negative for Runx1-GFP. (B and C) Runx1-GFP expression in c-Kit^+^ cells. (C) Quantitation of GFP^+^ cells from two embryos (35 sp). The frequency of Runx1-GFP^+^ cells in the head artery c-Kit^+^ cells is similar to that of circulating blood c-Kit^+^ cells. (D and E) Whole-mount immunostaining of E10.5 mouse embryos for c-Kit (green), CD31 (blue) and CD45 (magenta) expression. (D) Representative confocal images of the dorsal aorta and head region (36 sp). The arrowheads indicate c-Kit^+^CD45^+^ cells. (E) Quantitation of CD45^+^ cells from three embryos (36 and 37 sp). The frequency of CD45^+^ cells in the head artery c-Kit^+^ cells is similar to that of circulating blood c-Kit^+^ cells. Scale bars: 50 μm.

As shown in Fig 4A, Runx1-GFP is expressed in hemispherical cells, which are putative nascent hematopoietic cells at the wall of the dorsal aorta. However, the majority of c-Kit^+^ circulating cells in the lumen of the aorta were negative for Runx1-GFP (Fig 5A, arrowheads), indicating that nascent hematopoietic cells could be discriminated from circulating cells by the expression level of Runx1-GFP. Indeed, 92% of the c-Kit^+^ cells that were attached to endothelial cells in the dorsal aorta were Runx1-GFP^+^ whereas 39% of the c-Kit^+^ cells in the head arteries were Runx1-GFP^+^ (Fig 5B and 5C). This ratio was similar to that observed in circulating blood (32%), head veins (33%) and head capillaries (36%) (Fig 5C). These data suggest that the majority of c-Kit^+^ cells in the head arteries are not nascent hematopoietic cells but instead are circulating cells.

### CD45 expression in head c-Kit^+^ cells

To further characterize c-Kit^+^ cells in the head vasculature, we investigated the expression of CD45, which is a known marker of hematopoietic cells except erythrocytes and platelets. We previously showed that CD45 is expressed on the rim of hematopoietic clusters but not in the basal region (nascent hematopoietic cells) [9]. Therefore, similar to Runx1-GFP expression, nascent cells could be distinguished from maturating cells by the expression of CD45. In the dorsal aorta, 85% of the c-Kit^+^ cells that were attached to the endothelial layer were negative for CD45 (Fig 5D and 5E). In contrast, 43% of the c-Kit^+^ cells that were attached to the head arteries were negative for CD45 (Fig 5D and 5E). A similar frequency (51%) was also observed for the c-Kit^+^ circulating cells within the dorsal aorta (Fig 5E). These data strongly support the notion that the majority of c-Kit^+^ cells in the head arteries are circulating cells.

### Fate-mapping analysis of Runx1^+^ cells

**Fig 6:**
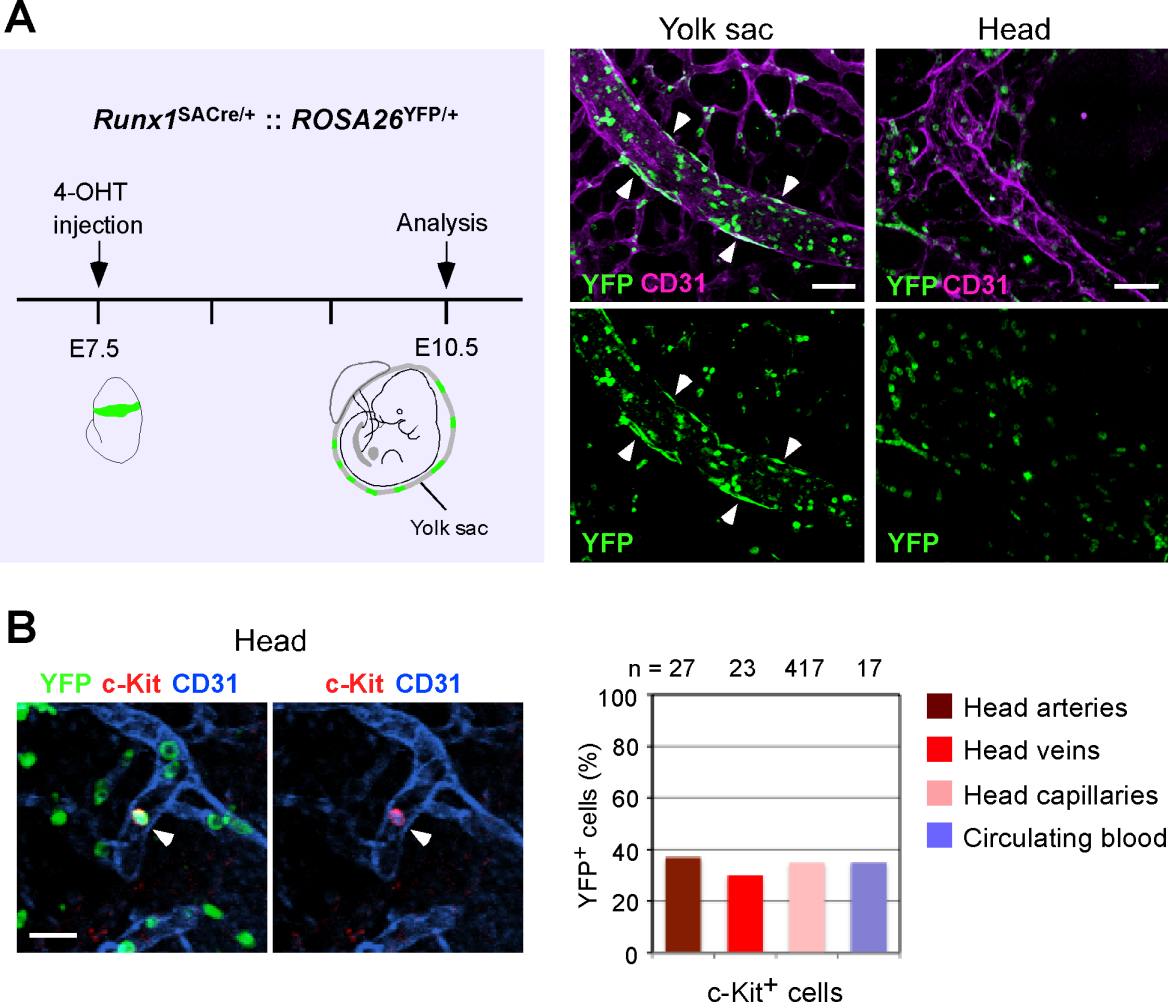
Majority of head artery c-Kit^+^ cells originate from other organs. (A) Experimental design for tracing the progenies of yolk sac hemogenic endothelium. *Runx1*^*SACre/+*^:: *ROSA26*^*YFP/+*^ embryos were labeled at E7.5 and analyzed at E10.5. Note that many YFP^+^ endothelial cells (arrowheads) were observed in the yolk sac but not in the head. Scale bar: 50 μm. (B) Representative confocal image of an YFP^+^ c-Kit^+^ cell (arrowhead) in the head. Scale bar: 20 μm. (C) Quantitation of YFP^+^ c-Kit^+^ cells from three embryos (36–38 sp).

Foregoing data suggest that the majority of c-Kit^+^ cells in the head arteries originate from other organs, most likely from the yolk sac endothelium [34]. To identify the contribution of other organs to head c-Kit^+^ cells, we took advantage of *Runx1*^*SACre/+*^:: *ROSA26*^*YFP/+*^ mice, which enabled us to trace the progenies of Runx1^+^ cells based on the expression of YFP [26]. An injection of 4-OHT at E7.5 labeled a large number of endothelial cells in the yolk sac at E10.5 (Fig 6A) [35]. In contrast, the head arteries were nearly devoid of YFP^+^ endothelial cells (0–6 arterial endothelial cells in the entire head, Fig 6A). These results suggest that the majority of YFP^+^c-Kit^+^ circulating cells are derived from the YFP^+^ yolk sac hemogenic endothelium. Based on these observations, we hypothesized that c-Kit^+^ cells generated from head arterial endothelial cells (YFP^-^cells) should be negative for YFP. Furthermore, the frequency of YFP^+^c-Kit^+^ cells that are localized in head arteries should be lower than that in other parts of the head vasculature. To assess the contribution of head arterial endothelial cells to c-Kit^+^ cells, we performed triple immunostaining (c-Kit, YFP and CD31). The head vasculature contained similar proportions of YFP^+^c-Kit^+^ cells in the arteries (37%), veins (30%), capillaries (35%) and circulating blood (35%) (Fig 6B), suggesting that, similar to c-Kit^+^ cells in the veins and capillaries, the majority of c-Kit^+^ cells in the head arteries originate from other organs.

## Discussion

Recent work has suggested that the embryonic head vasculature is involved in the de novo generation of HSCs [22]. To identify the hemogenic sites within the head region, we extensively analyzed hematopoietic clusters and the hemogenic endothelium in the entire head between E9.5 and E11.5. However, we did not identify apparent EHT structures in the head. Consistently, endothelial cell populations isolated from E10.5 heads rarely display hemogenic potential *in vitro* [17]. Taken together, these results suggest that the majority of c-Kit^+^ hematopoietic cells observed within the head are not derived from the head endothelium.

Although our immunostaining analyses did not reveal apparent EHT structures in the head, a small number of c-Kit^+^ cells may be generated from head arteries and may contribute to the adult HSC population. However, this possibility is unlikely if HSCs in the head and dorsal aorta are generated in the same manner. Our previous study revealed that 439 ± 87 c-Kit^+^ cells were localized on the wall of the dorsal aorta at E11.5 [9]. At this stage, HSC numbers have been estimated to be one per AGM [36], indicating that the efficiency of HSC generation within the aortic cluster is low (0.2%). In this study, we observed 25 ± 12 c-Kit^+^ cells in E10.5 head arteries (Fig 2). Because many c-Kit^+^ clusters are required to generate one HSC in the dorsal aorta, the number of c-Kit^+^ cells in the head arteries appears to be insufficient to generate HSCs.

The transcription factor Runx1 is essential for EHT and has been shown to be a marker of the hemogenic endothelium [18, 20, 21, 25]. We previously demonstrated that the *Runx1-GFP* transgene, in which GFP is driven by a *Runx1* +24 mCNE enhancer element, was induced in the endothelium of several hemogenic sites, including the dorsal aorta, the vitelline and umbilical artery, the yolk sac and the placenta [25]. In this study, we found that Runx1-GFP is not expressed in the head vasculature although it is expressed at other hemogenic sites. Although we cannot exclude the possibility that the transgene does not fully recapitulate the endogenous expression of Runx1 in the head, authentic hemogenic endothelial cells were likely absent in the head vasculature. An enhancer element is known to be activated in a cell-type specific manner, i.e., a context-dependent manner [37]. If the response of the enhancer element differs between two cell types, the cellular context differs between those cell types. Thus, we consider the cellular context of head endothelial cells to differ from that of Runx1-GFP^+^ hemogenic endothelium in other tissues.

Based on our study, HSCs may not be directly generated from the head endothelium, and external HSCs may not colonize the head. Live imaging or a more sophisticated lineage-tracing experiment may confirm this finding.

## Supporting Information

S1 Fig Sufficient penetration of antibody in the embryonic head. Whole-mount immunostaining of the E10.5 head for CD45 (green) and CD31 (magenta) expression. Note that CD45^+^ cells are scattered throughout the head. The thickness of the sample is shown in the y-z plane.
(TIF)

S2 Fig Characterization of c-Kit^+^ cells in the head mesenchyme. (A) Whole-mount immunostaining of the E10.5 head for c-Kit (green), CD45 (magenta) and CD31 (blue) expression. The arrows indicate mesenchymal c-Kit^+^ cells. These cells are CD45^-^CD31^-^. Scale bar: 50 μm. (B) FACS analysis of the E10.5 head region.
(TIF)

S3 Fig Number of c-Kit^+^ cells in the head vasculature at E11.5 (n = 4). (TIF)

S4 Fig Cartographic distribution of c-Kit^+^ cells in the head vasculature at E10.5 (35–36 sp). The grey region represents arteries. EC: endothelial cells.
(TIF)

S5 Fig Hematopoietic clusters at E9.5–11.5. (A) Number of c-Kit^+^ hematopoietic clusters with more than 10 cells in the dorsal aorta (E10.5, n = 3) and the whole head vasculature (E9.5, n = 2; E10.5 n = 4; E11.5, n = 4). One cluster was observed in the artery of E11.5 head (embryo no.4). DA: dorsal aorta. (B-E) Confocal image of c-Kit (green) and CD31 (magenta) expression. (B) Representative 3D image of E10.5 dorsal aorta. Scale bar: 100 μm. (C) Higher magnification view of boxed region in B. Scale bar: 50 μm. (D) 3D image of E11.5 head (embryo no.4). The whole-head image was acquired using tile scanning (49 tiles). Scale bar: 500 μm. (E) Higher magnification view of boxed region in D. Arrowhead indicates cluster localized in the artery. Although we could not determine the origin of this cluster, it is possible that it migrated from other organs via circulation, because we sometimes observed circulating large clusters in the lumen of dorsal aorta (not shown). Scale bar: 50 μm.
(TIF)
