# Supplementary figures and images for: Lack of Phenotypical and Morphological Evidences of Endothelial to Hematopoietic Transition in the Murine Embryonic Head during Hematopoietic Stem Cell Emergence
Source: PLoS One. 2016 May 26;11(5):e0156427. doi: 10.1371/journal.pone.0156427 (PMC4882078; doi:10.1371/journal.pone.0156427)

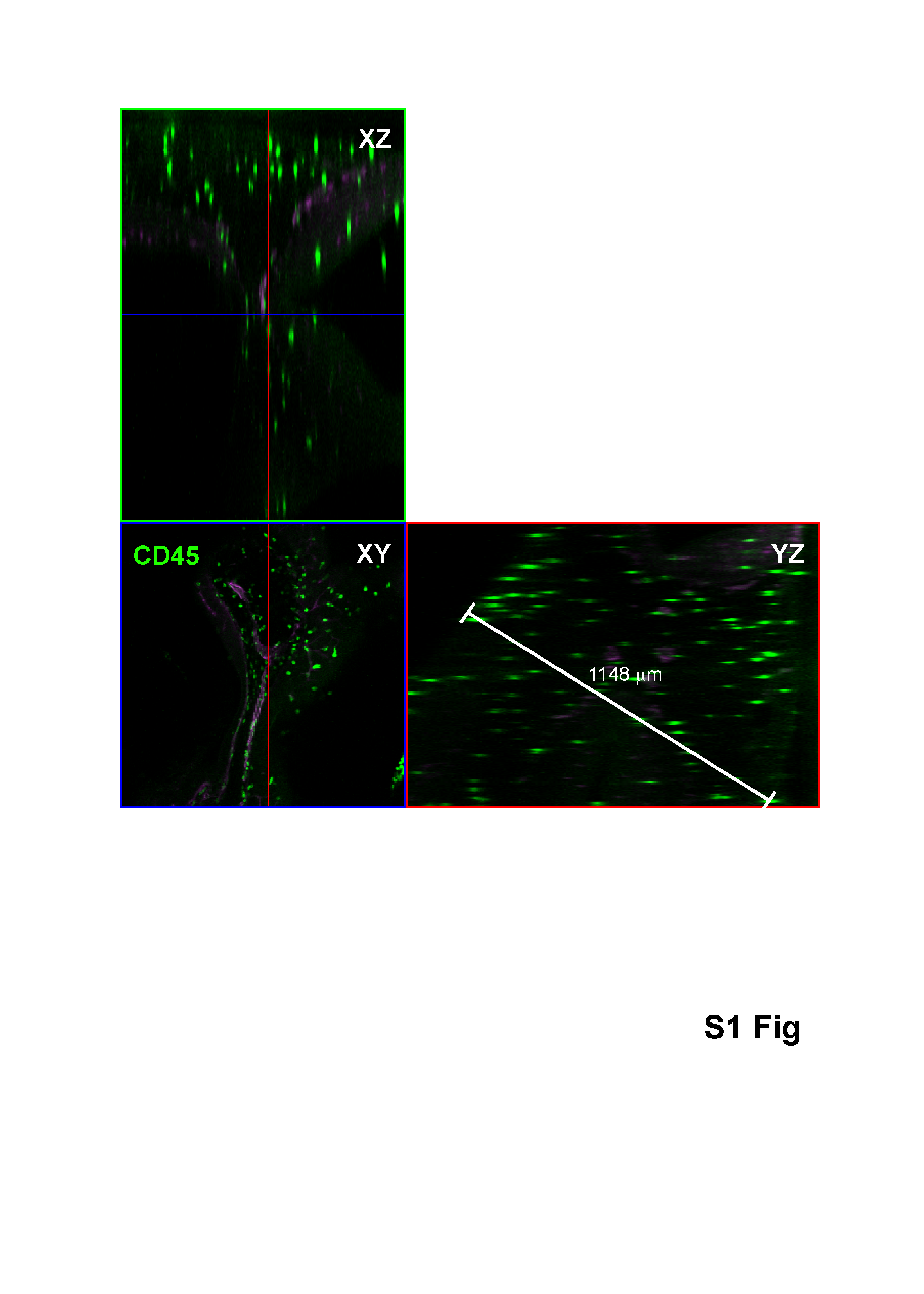

Supplement: S1 Fig — Whole-mount immunostaining of the E10.5 head for CD45 (green) and CD31 (magenta) expression. Note that CD45+ cells are scattered throughout the head. The thickness of the sample is shown in the y-z plane. (TIF) [file pone.0156427.s001.tif]

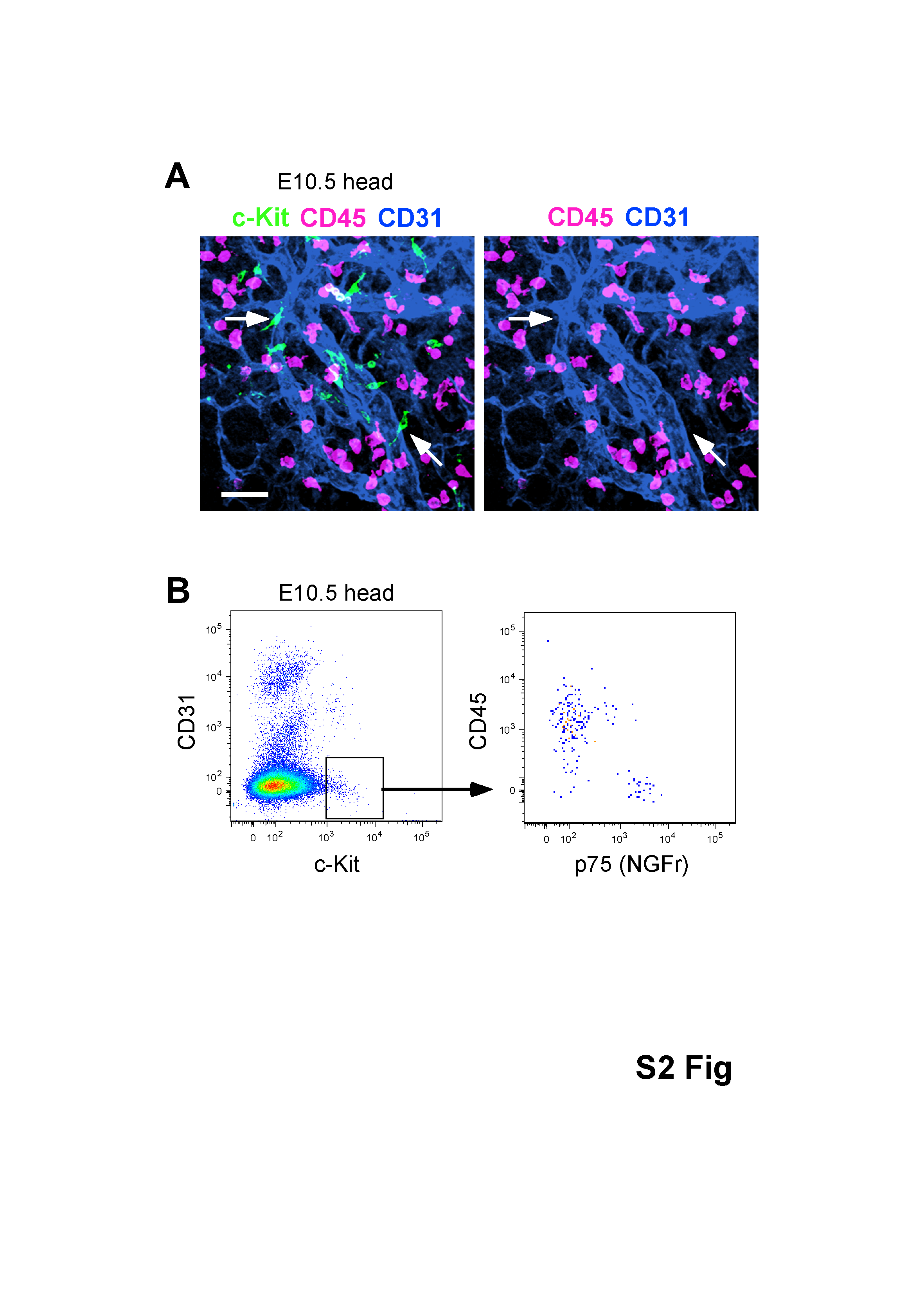

Supplement: S2 Fig — (A) Whole-mount immunostaining of the E10.5 head for c-Kit (green), CD45 (magenta) and CD31 (blue) expression. The arrows indicate mesenchymal c-Kit+ cells. These cells are CD45-CD31-. Scale bar: 50 μm. (B) FACS analysis of the E10.5 head region. (TIF) [file pone.0156427.s002.tif]

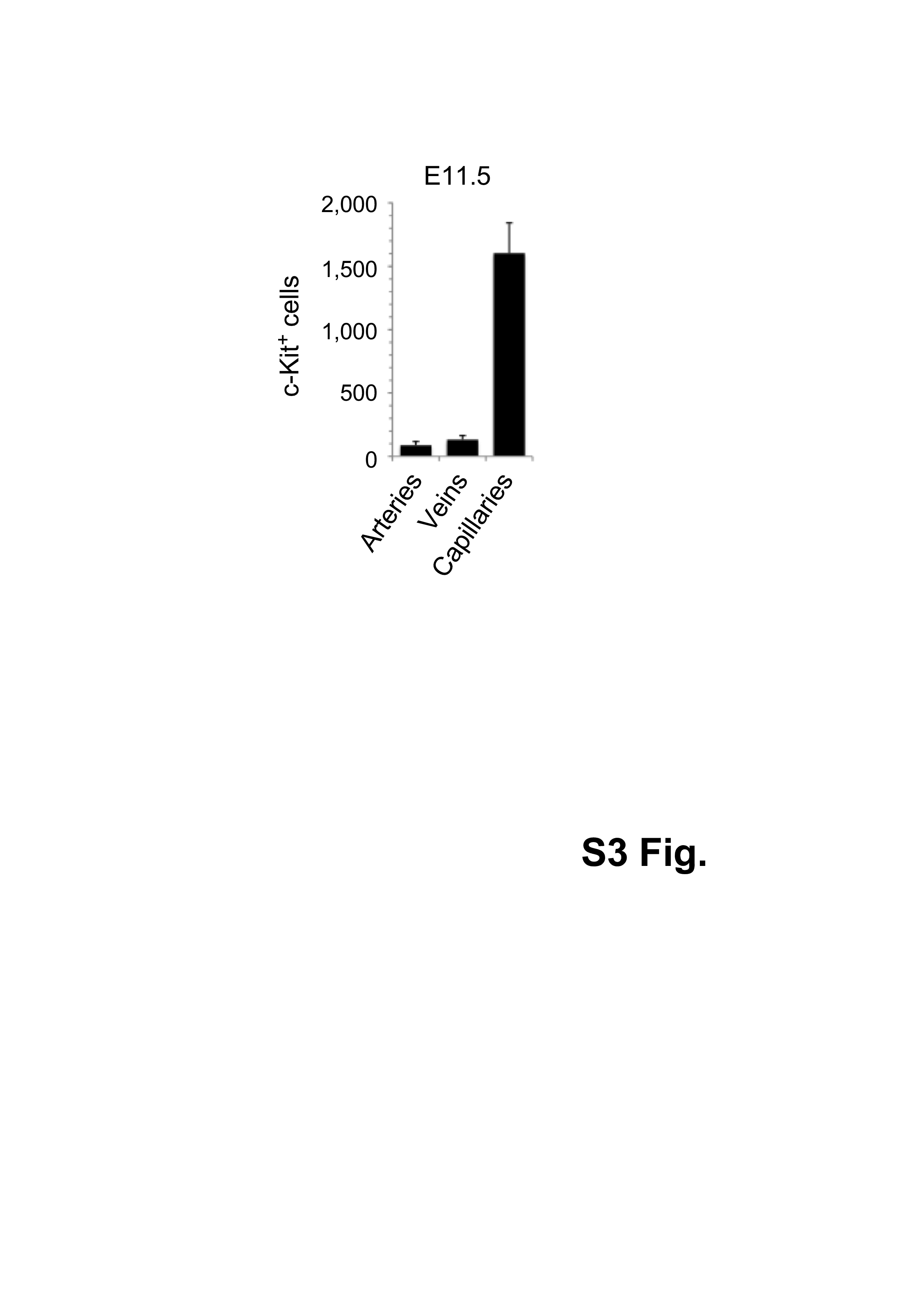

Supplement: S3 Fig — (TIF) [file pone.0156427.s003.tif]

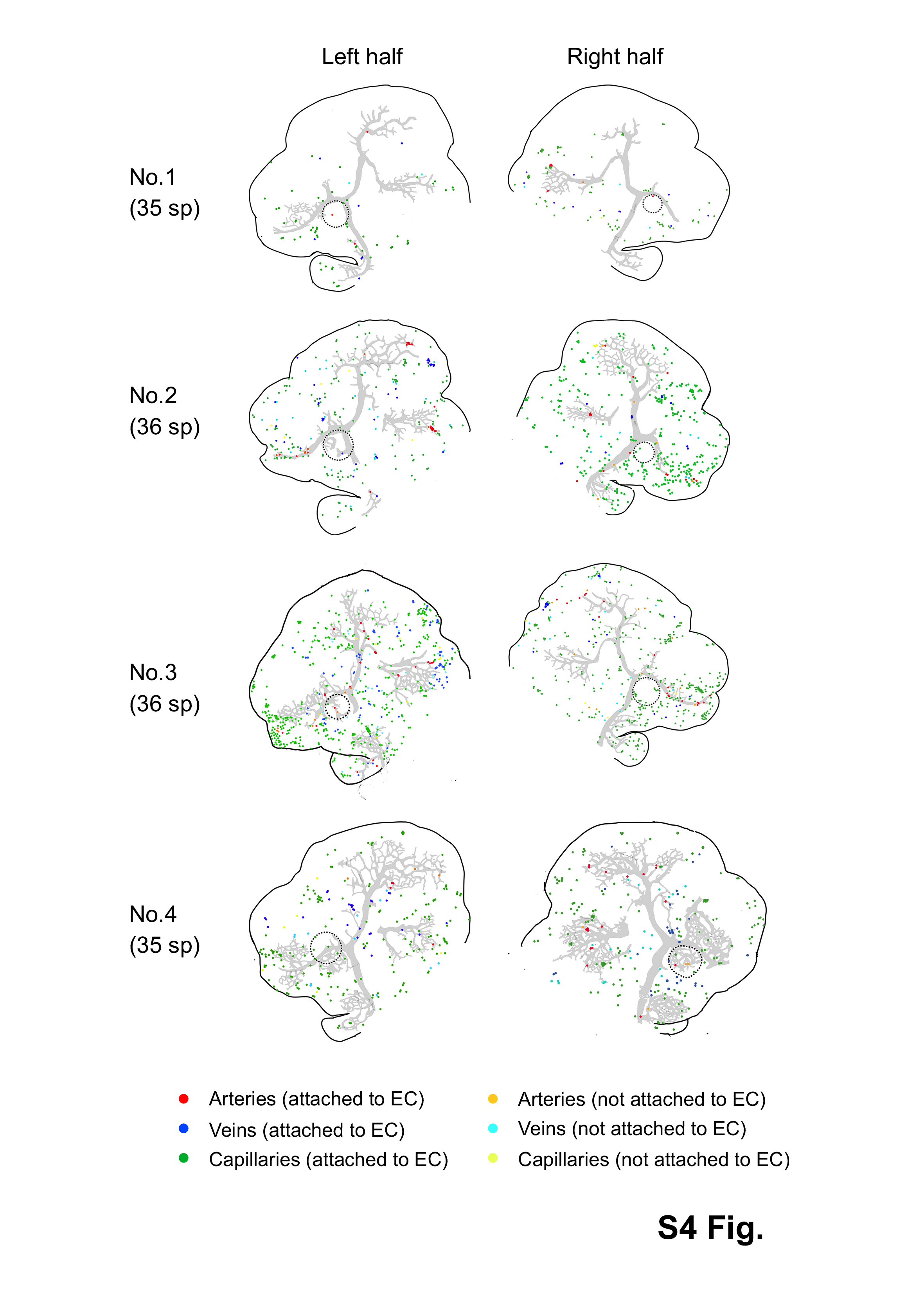

Supplement: S4 Fig — The grey region represents arteries. EC: endothelial cells. (TIF) [file pone.0156427.s004.tif]

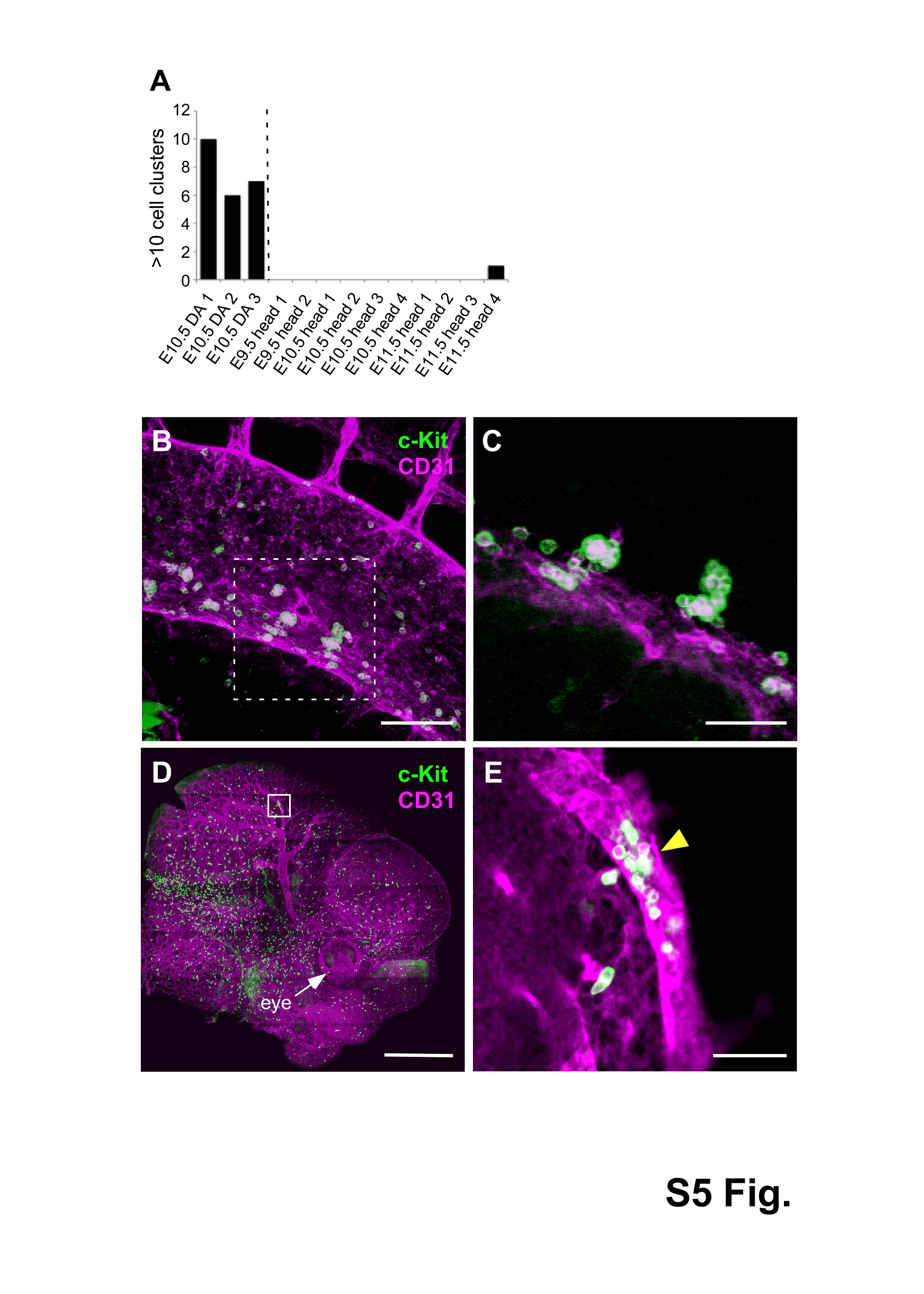

Supplement: S5 Fig — (A) Number of c-Kit+ hematopoietic clusters with more than 10 cells in the dorsal aorta (E10.5, n = 3) and the whole head vasculature (E9.5, n = 2; E10.5 n = 4; E11.5, n = 4). One cluster was observed in the artery of E11.5 head (embryo no.4). DA: dorsal aorta. (B-E) Confocal image of c-Kit (green) and CD31 (magenta) expression. (B) Representative 3D image of E10.5 dorsal aorta. Scale bar: 100 μm. (C) Higher magnification view of boxed region in B. Scale bar: 50 μm. (D) 3D image of E11.5 head (embryo no.4). The whole-head image was acquired using tile scanning (49 tiles). Scale bar: 500 μm. (E) Higher magnification view of boxed region in D. Arrowhead indicates cluster localized in the artery. Although we could not determine the origin of this cluster, it is possible that it migrated from other organs via circulation, because we sometimes observed circulating large clusters in the lumen of dorsal aorta (not shown). Scale bar: 50 μm. (TIF) [file pone.0156427.s005.tif]
